# Biomechanical features of a novel step-down-and-pivot task in football players with hip/groin pain

**DOI:** 10.1098/rsos.240908

**Published:** 2025-05-21

**Authors:** Prasanna Sritharan, Matthew G. King, Mario Andres Muñoz, Joshua J. Heerey, Mark J. Scholes, Benjamin F. Mentiplay, Adam I. Semciw, Joanne L. Kemp, Peter R. Lawrenson, Kay Crossley, Anthony G. Schache

**Affiliations:** ^1^ La Trobe Sports and Exercise Medicine Research Centre, La Trobe University, Melbourne, Victoria, Australia; ^2^ School of Computing and Information Systems, The University of Melbourne, Melbourne, Victoria, Australia; ^3^ ARC Industrial Transformation Training Centre in Optimisation Technologies, Integrated Methodologies, and Applications (OPTIMA), Australian Research Council, Carlton, Victoria, Australia; ^4^ School of Health and Rehabilitation Sciences, The University of Queensland, St Lucia, Queensland, Australia

**Keywords:** femoroacetabular impingement, principal component analysis, biomechanics, feature selection, gait analysis, hip osteoarthritis

## Abstract

Identifying biomechanical impairments in young, physically active populations with hip/groin pain is crucial for early intervention. This study characterized the biomechanical features of a novel task, the *step-down-and-pivot*, in competitive football players, comprising 36 individuals with hip/groin pain (28 ± 6 years) and 11 controls (26 ± 4 years). Experimental motion data and ground forces were input into biomechanical models to calculate joint angles and moments, then transformed into principal components and input into a feature selection pipeline. Ten main biomechanical features were identified for each limb, i.e. the pivot limb and the swing limb, that could discriminate between symptomatic and control groups with *p* < 0.05. In symptomatic individuals, the main features were as follows: *pivot limb*: smaller hip flexion and knee extension angles, delayed initiation of hip flexion and increased ankle dorsiflexion moment; *swing limb*: reduced hip flexion moment, increased hip internal rotation moment, delayed hip adduction and knee extension moments, and reduced ankle dorsiflexion angle. The largest group differences occurred during the transitions from step-down to pivot, and pivot to step-forward, supporting a potential role for multi-phase and/or multi-planar tasks when assessing biomechanical impairments due to hip/groin pain. Although biomechanical alterations in our symptomatic participants were small, they could be identified and characterized using feature selection.

## 1. Introduction

Hip and/or groin (hip/groin) pain is common in physically active young- to middle-aged adults, and is associated with reduced physical function, decreased participation in sports and reduced quality of life [1,2]. One study of 695 sub-elite footballers found that 49% of players experienced hip/groin pain during their previous season, with 31% reporting pain for greater than 6 weeks [1]. Pathological conditions associated with the hip joint, such as femoroacetabular impingement syndrome, labral tears and chondral lesions, are frequent clinical findings in athletic populations with hip/groin pain [2–5]. These individuals may have a two-to-ninefold elevated risk of developing future hip osteoarthritis [6], with the prevalence of radiographic hip osteoarthritis in retired middle-aged elite soccer players and handballers exceeding 50% [6,7]. Therefore, early and appropriate identification and intervention in these athletic populations is crucial.

Studies have shown that hip/groin pain may be associated with altered movement kinematics and kinetics (hereafter simply referred to as ‘biomechanics’), which could be used to identify individuals at risk of future worsening of their condition. However, unidirectional level gait, including walking and running, seem inadequate for this purpose, as studies of such tasks have found few differences compared with asymptomatic controls [8–10]. Tasks that load the hip more, such as single-leg drop landings [11,12], drop jumps [10], lateral hop tests [13,14], lateral cutting [15,16] and stair ascent [17], might better distinguish between symptomatic and control biomechanics. While some studies of such tasks have reported notable kinematic and kinetic adaptations at the hip and pelvis [11,13,14], other studies have not [10,18,19], thus tempering their discriminatory power. Notably, two studies of sprinting with femoroacetabular impingement syndrome, in which hip joint loads exceeded 10 body weights, found limited differences in lower-limb joint kinematics and kinetics [20,21]. One proposed hypothesis for these findings of limited biomechanical differences despite high hip loads is that well-trained participants were more resilient to pain and thus able to undertake the tasks with good functional performance [10]. Alternatively, passive pain-provocation tests such as flexion-adduction-internal rotation (FADIR), while demonstrating good sensitivity, have low specificity for hip/groin pain conditions [22]. Thus, there is a need to identify suitable discriminatory movement tasks for early identification of biomechanical impairments associated with hip/groin pain in physically active adults, whose symptoms may initially be mild. To this end, we developed a novel multi-phase multi-directional movement task, the *step-down-and-pivot*, in which a participant steps down from a raised platform, pivots left or right, and then walks forward, reminiscent of turning to the left or right after descending a flight of stairs [23]. We proposed that this task would integrate moderately high hip-joint loading during the step-down phase, akin to stair descent [24], with the combined movements of hip flexion, adduction and internal rotation during the pivot phase, thus potentially overcoming some of the limitations of the routine tasks and passive tests described earlier.

Nevertheless, it is possible that small impairments may be an embedded characteristic of younger physically active adults with hip/groin pain, regardless of the task undertaken. Therefore, a second challenge for identifying biomechanical impairments in this population is the development of suitable analytical approaches that can detect and describe differences between symptomatic and asymptomatic groups that may not have practical consequences in the early stages of hip/groin pain, but may reflect future risk of developing serious musculoskeletal conditions such as hip osteoarthritis To this end, principal component analysis (PCA), which has seen widespread application in musculoskeletal science, is well-suited to the analysis of features in biomechanical waveform data [25]. Only three studies have applied PCA to biomechanical analyses of the symptomatic hip. Rutherford *et al*. [8] applied PCA to evaluate the utility of level walking for studies of femoroacetabular impingement syndrome and were able to identify and explain salient differences in gluteus maximum and hamstrings muscle activations despite considerable variability in activation patterns and no meaningful differences in biomechanics compared with asymptomatic controls. Meyer *et al*. [26] and Roach *et al*. [27] applied it together with linear discriminant analysis to identify the biomechanical features of hip osteoarthritis with the greatest discriminatory power, finding components of hip and knee extension angles and hip flexion and internal rotation moments best able to distinguish between osteoarthritic and healthy walking gait.

PCA transforms the original data into a set of unbiased, non-redundant features known as principal components (PCs) that each account for a proportion of total variance in the original variables [28]. Although PCs are mathematically abstract constructs, they can be meaningfully interpreted in the context of movement analysis by a domain expert inspecting results [28]. To identify features that distinguish biomechanical impairments in the presence of pathology, PCA is typically followed by statistical hypothesis testing [29–31], classification [26,31–33] or regression analyses [34]. Importantly, PCA-based classification approaches can isolate and amplify subtle group differences [31], reaching accuracy, sensitivity and specificity as high as 0.94, 0.96 and 1.00, respectively [35]. Thus PCA-based approaches have demonstrated excellent discriminatory power, and can help qualitatively describe the modes of variation that distinguish between groups, such as differences in joint range of motion or timing of muscle activation.

The objective of this exploratory study was to quantify and compare joint angles and moments in a cohort of young adult football players with hip/groin pain versus asymptomatic controls when undertaking the step-down-and-pivot task. To achieve this objective, we applied a previously published feature selection process [31], based on PCA and statistical learning methods, to identify the salient features of joint angles and moments for the step-down-and-pivot task that could best discriminate between symptomatic individuals and asymptomatic controls, and to qualitatively describe the modes of variation that distinguished the groups.

## 2. Methods

### 2.1. Study design and participants

Thirty-six individuals with hip/groin pain and 11 asymptomatic controls, age 18−50, participated in this cross-sectional study (table 1). Due to the exploratory nature of our study, no formal power analysis was conducted to determine sample sizes *a priori*. Participants were competitive soccer players, completing at least two formal sessions per week, and were a subset of the larger Femoroacetabular Impingement and Hip Osteoarthritis cohort (FORCe) study investigating longitudinal changes in hip structure and symptoms in football players (both Australian Rules football and soccer) [23]. Ethical approval was obtained from the La Trobe University Human Ethics Committee (HEC015-019 and HEC016-045).

**Table 1.** Participant information and patient-reported outcome measures for symptomatic hip/groin pain and control groups.

	**symptomatic** **(*****n*** **= 36)**	control (*n* = 11)	*p*
age, years	28 (6)	26 (4)	0.668
sex, male/females (% female)^a^	25/11 (30%)	9/2 (18%)	0.422
weight, kg	73.7 (13.0)	75.2 (14.3)	0.762
height, m	1.75 (0.09)	1.77 (0.07)	0.367
bilateral pain, no. of participants (% bilateral)	26 (72%)	—	—
pain level over previous week, range 0−10^b^	3.5 [2]	—	—
patient-reported outcome measures, range 0−100^c^^,^^d^			
iHOT-33 total	68.3 [22.3]	99.7 [3.2]	<0.001
HAGOS symptoms	58.9 [15.1]	100 [0.0]	<0.001
HAGOS pain	75.0 [14.4]	100 [0.0]	<0.001
HAGOS physical function in daily living	80.0 [22.5]	100 [0.0]	<0.001
HAGOS function in sports and recreation	64.0 [20.3]	100 [0.0]	<0.001
HAGOS participation in physical activities	50.0 [37.5]	100 [0.0]	<0.001
HAGOS quality of life	60.0 [26.3]	100 [0.0]	<0.001

Data presented as mean (standard deviation) or median [interquartile range] as appropriate. Groups compared using Welch’s *t*‐test unless indicated otherwise.

^a^ Groups compared with *χ*^2^ test

^b^ Pain level scales are 0 (no pain) to 10 (extreme pain)

^c^ Groups compared with Mann–Whitney *U* test

^d^ HAGOS and iHOT-33 scales are 0 (extreme pain) and 100 (no pain). iHOT-33: International Hip Outcome Tool 33. HAGOS: Copenhagen Hip and Groin Outcome Score

Participants provided written informed consent obtained prior to participation. Inclusion and exclusion criteria are extensively detailed in the FORCe protocol description [23]. Briefly, symptomatic participants needed to report more than six months of activity-related hip/groin pain and have a positive hip rotation pain provocation (FADIR) test. Asymptomatic control participants reported no history of hip/groin pain and had a negative pain provocation test. Participants quantified their average pain level in the prior 7 days using a numerical scale (0−10). Self-reported hip/groin pain burden was quantified using the International Hip Outcome Tool 33 [36] (iHOT-33) and Copenhagen Hip and Groin Outcome Score [1] (HAGOS).

### 2.2. Biomechanical experiment and data collection

**Figure 1.**
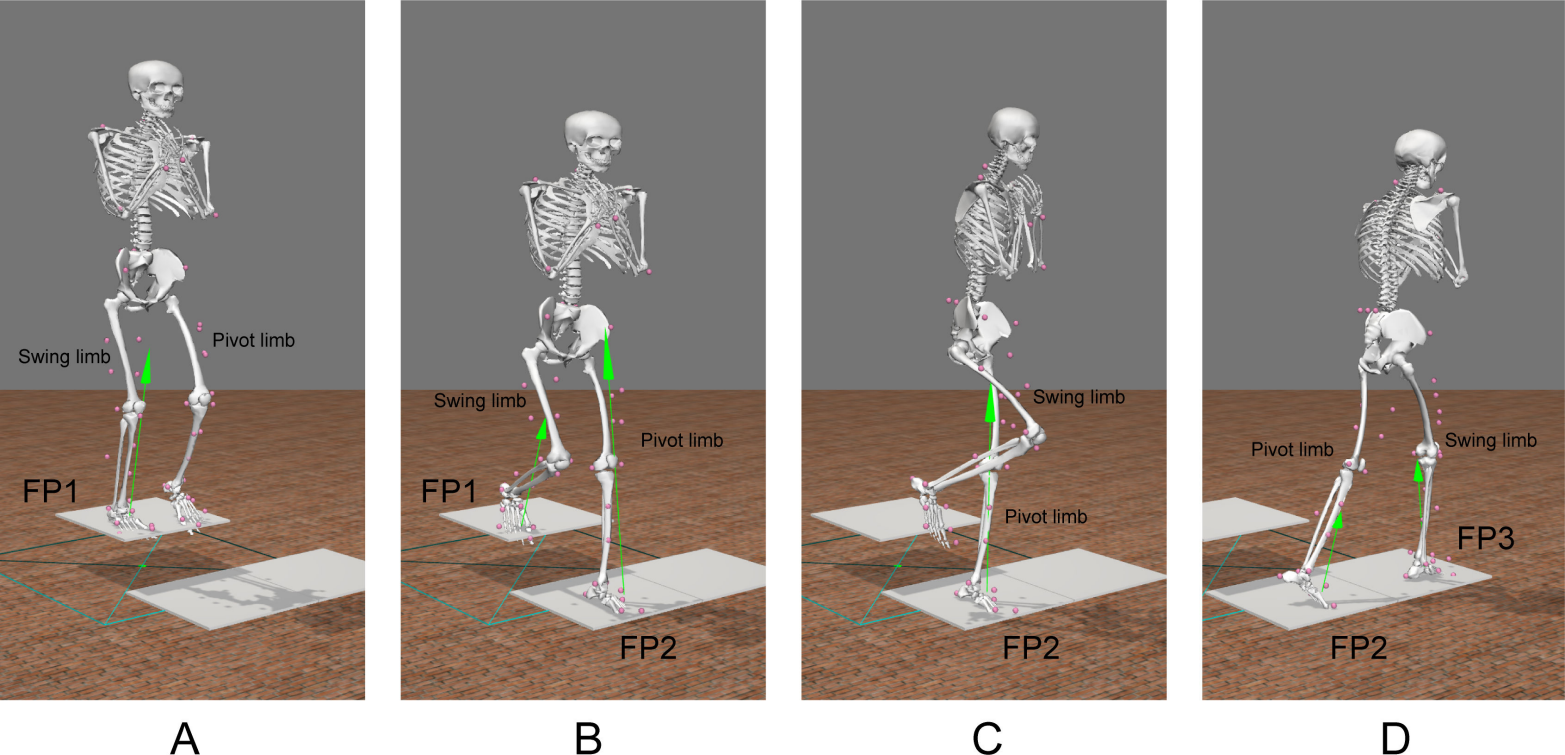
The step-down-and-pivot task with left limb as the *pivot limb* and right limb as the *swing limb*. (A) The participant stands on the step, located 20 cm above the ground. (B) The participant steps down, with the pivot limb striking the force plate FP2 on the ground, and the swing limb remaining on FP1. (C) The participant initiates a 90° counter-clockwise pivot with the pivot limb, during which time the swing limb is lifted from FP1. (D) The swing limb swings around to strike the additional force plate on the ground FP3 before the participant steps forward by lifting the pivot limb from FP2.

The step-down-and-pivot task consisted of three movement phases undertaken in one single continuous action: (1) step-down; (2) pivot; and (3) step-forward (figure 1). Each participant initially stood with both feet on the force plate on the step (FP1), which was located 20 cm above the ground (figure 1A). One leg steps down and is the *pivot limb*; the other is the *swing limb*. The sequence of phases for a trial is described as follows, with the left limb as the pivot limb, the right limb is the swing limb, and FP1 positioned behind FP2:

(1) *Step-down*. The participant steps down, with the left limb striking the force plate on the ground (FP2), and the right limb remaining on FP1 (figure 1B).
(2) *Pivot*. The participant initiates a 90° counter-clockwise pivot with the left limb, during which time the right limb is lifted from FP1 (figure 1C), and swings around to strike the additional force plate on the ground FP3 (figure 1D).
(3) *Step-forward*. Finally, the participant initiates a step forward by lifting the left limb from FP2.

A total of 10 step-down-and-pivot trials were undertaken for each participant at their own self-selected pace, the first five in which the right limb was the pivot limb with the pivot direction clockwise, and the subsequent five in which the left limb was the pivot limb with the pivot direction counter-clockwise. For trials where the right leg was the pivot limb, FP1 was repositioned behind FP3, and participants stepped down to FP3 then pivoted clockwise towards FP2.

Kinematic data, specifically the trajectories for 46 retro-reflective markers attached to the participants, were collected with a 10-camera opto-reflective motion capture system (Vicon, UK), sampling at 100 Hz. Ground reaction force data were collected using three force plates (AMTI, USA) sampling at 1000 Hz: two 400 × 600 mm plates embedded in the ground (i.e. FP2 and FP3, figure 1); and one 500 × 500 mm plate placed onto the raised step 20 cm above the ground (i.e. FP1, figure 1). Both kinematic and ground reaction force data were filtered at 10 Hz prior to subsequent analysis. Participants wore loose-fitting shorts (and a crop top for women) as well as Teva Original Universal sandals (Deckers Brands, USA). Prior to undertaking the task, participants completed a static calibration trial. The 46 retroreflective markers (with an additional four markers for static calibration trials only) were attached to specific landmarks on the participant’s lower limbs, pelvis, torso, neck and upper extremities as per our previously published protocol for this cohort [23] (reproduced in electronic supplementary material, figure S1).

### 2.3. Biomechanical modelling

Biomechanical modelling was performed with OpenSim 4.3 [37] via the application programming interface (API) using Python 3.8 (Python Software Foundation, python.org). A musculoskeletal model for each participant was generated by scaling a generic whole-body 22-segment 37-degree-of-freedom model, which has been previously validated by the model’s authors, Rajagopal *et al*. [38].

Rigid bodies were used to represent the pelvis, the combined head and torso as well as each femur, patella, tibia/fibula, talus, calcaneus, forefoot, humerus, ulna, radius and hand. Each knee, ankle, subtalar, metatarsophalangeal, elbow and radioulnar joint was modelled as a pin-joint aligned along their respective generic anatomical axes; the hips, lumbosacral joints and glenohumeral joints were modelled as ball-joints; and each wrist was modelled as a universal joint. For the present study, the wrist and subtalar joints were locked at their reference positions.

For each participant and recorded trial, joint angles were calculated using an inverse kinematics approach that minimized the weighted sum of squared distances between model and experimental marker trajectories [39]. Internal joint moments were calculated using inverse dynamics by applying the joint kinematics and measured ground forces to the model.

### 2.4. Descriptive analysis and inference

In our present study, we analysed the pivot and swing limbs (figure 1) separately. Thus, we undertook two independent between-group analyses: (i) when the ‘reference’ limb was the pivot limb; and (ii) when the ‘reference’ limb was the swing limb. For each ‘reference’ limb, the groups compared were the more symptomatic limb for the hip/groin pain group (hereafter referred to simply as symptomatic limbs) and both limbs for the asymptomatic group (hereafter simply referred to as control limbs). For the purposes of our descriptive analysis, data from the left and right control limbs were averaged together.

The biomechanical variables of interest were joint angles (denoted by *θ*) and joint moments (denoted by *M*) from eight coordinates defined in our biomechanical models: *HIPFLEX*, hip flexion; *HIPADD*, hip adduction; *HIPROT*, hip internal rotation; *KNEEEXT*, knee extension; *ANKLEDF*, ankle dorsiflexion; *LUMBAREXT*, lumbosacral extension; *LUMBARBEND*, lumbosacral lateral bending; and *LUMBARROT*, lumbosacral rotation. All temporal waveforms were resampled to 101 samples. Joint moments were normalized to units of *% of body weight × height* (%BW*HT). To compare group differences (more symptomatic limb versus control limb), we applied a two-sample *t*‐test with statistical parametric mapping (SPM) using the *spm1d* package (v. 0.4.18, spm1d.org) in Python [40]. As the 16 variables analysed per limb were non-independent, statistical significance was set at the Bonferroni-corrected level of α=0.003125. Group means and standard deviations of the waveform data were also calculated. Group demographic characteristics were summarized using mean and standard deviation, or median and interquartile range, as appropriate, using visual inspection of box plots and normality based on Shapiro–Wilks tests, and analysed using Mann–Whitney *U*-tests, independent sample *t*-tests, or *χ*^2^ tests, as required with the *SciPy* package (v. 1.10.1) in Python.

### 2.5. Feature selection, analysis and inference

To identify the features of the step-down-and-pivot task that differed between the two groups, we applied a previously developed biomechanical feature selection process [31] using MATLAB 2022a (The Mathworks, Natick, MA) based on weighted PCA. This process has already been extensively mathematically detailed [31], and is only briefly described here.

A total of 300 step-down-and-pivot trials across all participants and limbs tested (193 trials for the symptomatic limb and 107 trials for the control limb) were input into the feature selection process. Note, for the control group, all available trials for both the left and right limb were input into the pipeline, i.e. the left and right limb trials were not averaged. All trials from symptomatic and control groups were pooled together, weighted and transformed from 16 time-varying biomechanical variables (eight joint angles and eight joint moments each with 101 temporal samples) into a set of new variables, or features, known as principal components (PCs). A normalized weighting regime was applied to each trial based on the total number of trials recorded for that participant as well as the total trials for each other participant as previously described by Sritharan *et al*. [31] To simplify our feature analysis, we specifically considered only the variables associated with the ‘reference’ limb (pivot or swing) and the lumbosacral joint. Variables associated with the contralateral limb and upper extremity were excluded to reduce the analytical complexity.

Each PC is a unit eigenvector of the weighted correlation matrix of the original data, and is a vector of *coefficients* that describe a linear combination of the original variables. Its corresponding eigenvalue describes the variance explained by each PC. The PC analysis transforms every temporal sample from each waveform of the original data into a signed value called its *score*, that describes how far a specific temporal sample deviates from its PC, which by definition has a score of zero. From the PC scores for each temporal sample, we can calculate mean PC scores for a single trial by averaging the PC scores for a single waveform, and also for groups of trials (e.g. our symptomatic and control groups) by averaging the mean PC scores for all the trials in the group. The set of PCs is ordered, with the first PC, i.e. PC1, explaining the most variance in the original data. In our present study, we arbitrarily define ‘high’ PCs as PC1−3, and ‘low’ PCs as PC4 onwards.

For each ‘reference’ limb, we applied three statistical learning analyses in sequence to systematically reduce the number of PCs to a final set of 10 PCs, or *main features*, that could best distinguish between symptomatic and control biomechanics for the task:

(1) *Parallel analysis* [41]. Only those PCs that explained most of the variance in the data were retained. That is, given a set of *p* PCs, if most of the variance in the data was explained by the first *k* PCs, the remaining p-k components were dropped. Specifically, non-trivial PCs were defined as having eigenvalues larger than PCs derived from random data with the same sample size and number of variables. The number of retained PCs were 164 and 148 for the pivot and swing limb, respectively (electronic supplementary material, tables S1 and S2).
(2) *Weiss–Indurkhya independent features selection* [42]. For each retained variable, the mean PC scores were calculated for the symptomatic and controls groups, and compared using Welch’s *t*‐test. Only PCs for which the between-group score comparison returned t≥2.0 (approximately, α=0.05) were kept, resulting in 49 and 47 retained PCs for the pivot and swing limb, respectively (electronic supplementary material, tables S1 and S2).
(3) *Sequential feature selection* [43]. The remaining PCs were successively added to an empty set until the addition of further PCs did not improve the stopping criterion, implemented as the 10-fold cross-validated misclassification rate of a naive Bayes classifier, or the number of valid features, set to 10, was exceeded. As each iteration of sequential feature selection could produce different results due to the random nature of the cross-validation, 1000 iterations were performed, with the 10 most-frequently selected PCs retained, forming a final set of main features for each respective scenario (electronic supplementary material, tables S1 and S2).

Group differences in PC scores for each main feature were evaluated using Welch’s *t*-tests at a narrower significance level of α=0.001, as these features were already significantly different at α=0.05. PC scores were reported as mean and standard deviation. Effect sizes were calculated using Hedge’s *g*.

### 2.6. Associated features

Sequential feature selection excludes PCs that are well-correlated with the main features if they do not improve model accuracy. However, these *associated features* may be important in understanding biomechanical differences between groups. Therefore, for each main feature, the Pearson correlations ρ between it and every other remaining PC after parallel analysis were calculated. We defined *associated features* as those remaining PCs that correlated moderately (0.5≤|ρ|<0.7) or strongly (|ρ|≥0.7) with their respective main feature at significance level α=0.05, based on ranges suggested by Hinkle *et al*. [44]. We further defined *significant associated features* as those associated features for which the group differences in PC scores were significant at level t≥2.0 (approximately, p<0.05), analysed using *t*-tests for consistency with Weiss–Indurkhya independent features selection.

### 2.7. Interpretation of features

PCs are abstract constructs, and to be meaningful, must be qualitatively interpreted within their applied context. To do this, for each main feature, we undertook the following:

(1) To understand how each feature (i.e. each PC in the final feature sets) modulated its associated biomechanical variable, we plotted the waveforms of the original variable that corresponded to the upper and lower quartiles of PC scores for that feature (see figures 4 and 5, top row). The upper quartile waveform represented a trial with a large positive PC score, while the lower quartile waveform represented a trial with a large negative PC score. By presenting two waveforms of the original biomechanical variable representing PC scores sufficiently distanced either side of the pooled mean PC score (which always has a score of zero), the effect of the PC on the variable could be clearly and qualitatively described. Note that we have not yet considered the group (symptomatic or control).
(2) As our biomechanical variables are temporal in nature, a feature may influence the variable during particular time periods during the task. To understand the time periods during the task in which the PC had greatest influence, we plotted the PC coefficient vector, as well as the variance of the original data explained by that feature at any time instant, i.e. the vector of squared correlations between each temporal sample of the original data and the PC (see figures 4 and 5, bottom row). Time windows during which both the coefficient and squared correlation were large, i.e. the magnitudes of both were closer to 1, corresponded to time periods during the task where the PC had the greatest influence on the waveform of the original biomechanical variable. During time periods where the magnitudes of either or both were closer to zero, the PC had little influence on the waveform of the original biomechanical variable.
(3) Now consider the groups (symptomatic versus control). For a given feature, from the sign of the mean PC scores for each group (see table 2), we determined whether the symptomatic group tended towards the upper quartile (positive PC score) or lower quartile (negative PC score) of pooled PC scores for that feature. The control group must necessarily have a mean PC score of opposite sign and therefore tended towards the opposite quartile compared with the symptomatic group. Thus, we could interpret and describe the effect of the PC on the biomechanical variable for each group based on the quartile of PC scores to which it was closest (see table 3). This approach enabled us to qualitatively describe the effect of a PC on each group where magnitude of each group’s PC score was small, i.e. the between-group differences in PC scores were also small. In this case, the effect of the PC on each group would be described by the respective quartile to which it was closest, but the magnitude of the effect would be smaller if the magnitude of the mean PC score of the group was smaller than its associated quartile.
(4) For a given feature, by (i) comparing the waveforms of the biomechanical variable that represented the upper and lower quartiles of pooled PC scores and ascribing the symptomatic and control groups to the appropriate quartile (see figures 4 and 5, top row); (ii) identifying the time windows during the task in which the PC had greatest effect (see figures 4 and 5, bottom row), we could qualitatively describe the *modes of variation* that distinguish between symptomatic and control groups, e.g. temporal delays or differences in peaks and/or ranges of motion (see table 3). For example, for a particular PC, a negative PC score for the symptomatic group may indicate that the effect of that PC is to decrease a joint’s range of motion within a specific time window during the task, while a positive PC score for controls may represent an increased joint range of motion within that same time period.

**Table 2.** Group means and standard deviations of the principal component scores for the final selected feature sets for the pivot and swing limb during the step-down-and-pivot task.

feature	principal component scores		mean diff [95% CI]	*p*	*g*
	symptomatic	control			
pivot limb					
***θ_HIPFLEX_* PC1**	−5.85 (66.2)	25.4 (49.4)	−31.3 [−42.9, −19.671]	***<0.001***	−0.514
***θ_HIPFLEX_* PC3**	−0.981 (22.3)	6.09 (26.0)	−7.07 [−11.9, −2.246]	0.014	−0.298
*θ_HIPFLEX_* PC5	−1.48 (14.0)	6.43 (17.1)	−7.92 [−11.0, −4.803]	***<0.001***	−0.520
*θ_HIPFLEX_* PC6	0.453 (12.0)	−2.53 (9.24)	2.99 [0.853, 5.12]	0.027	0.268
*θ_HIPADD_* PC4	−1.05 (11.6)	4.02 (10.8)	−5.07 [−7.30, −2.84]	***<0.001***	−0.447
***θ_KNEEEXT_* PC1**	5.18 (43.7)	−17.7 (76.3)	22.9 [10.5, 35.3]	0.001	0.398
*M_KNEEEXT_* PC5	−0.348 (2.32)	1.29 (2.56)	−1.64 [−2.12, −1.15]	***<0.001***	−0.680
***M_ANKLEDF_* PC1**	−1.17 (7.66)	4.32 (8.47)	−5.50 [−7.11, −3.89]	***<0.001***	−0.689
*M_ANKLEDF_* PC5	−0.151 (3.52)	0.853 (3.72)	−1.00 [−1.73, −0.284]	0.021	−0.280
*M_LUMBARROT_* PC6	0.133 (1.15)	−0.552 (1.51)	0.685 [0.417, 0.952]	***<0.001***	0.530
swing limb					
*θ_HIPADD_* PC4	−0.708 (11.0)	3.51 (12.2)	−4.22 [−6.53, −1.91]	0.002	−0.369
***θ_ANKLEDF_* PC2**	−1.44 (25.1)	4.48 (22.9)	−5.92 [−10.7, −1.13]	0.043	−0.242
***M_HIPFLEX_* PC2**	−0.493 (6.31)	2.17 (7.57)	−2.66 [−4.05, −1.27]	0.001	−0.393
*M_HIPFLEX_* PC8	0.181 (1.99)	−0.579 (2.66)	0.760 [0.29, 1.23]	0.005	0.338
***M_HIPADD_* PC3**	0.294 (3.83)	−1.00 (4.86)	1.30 [0.42, 2.17]	0.010	0.307
*M_HIPADD_* PC10	−0.119 (1.52)	0.476 (2.05)	−0.595 [−0.954, −0.235]	0.004	−0.345
***M_HIPROT_* PC1**	0.353 (2.28)	−1.19 (3.51)	1.55 [0.956, 2.14]	***<0.001***	0.559
***M_KNEEEXT_* PC2**	−0.355 (6.40)	1.78 (6.49)	−2.13 [−3.42, −0.850]	0.007	−0.331
*M_ANKLEDF_* PC6	−0.116 (2.78)	0.421 (1.87)	−0.537 [−1.01, −0.066]	0.073	−0.214
*M_ANKLEDF_* PC9	0.0918 (1.20)	−0.367 (1.40)	0.459 [0.200, 0.718]	0.003	0.361

Principal component scores presented as mean (standard deviation) to three significant figures.

Mean difference [95% confidence interval (CI)] presented to three significant figures.

PC abbreviates the term ‘principal component’, and the numeric suffix is the number of that principal component, e.g. read PC3 as ‘*third principal component*’.

High principal components (i.e. PCs 1−3) are named in **bold**.

*p*-values calculated using *t*‐test between symptomatic and control groups at an *a priori* significance level of α=0.001 as the features input into sequential feature selection were already significant at t≥2.0 (approx. α=0.05).

Significant *p*-values are presented in ***bold italic***.

Hedge’s *g* effect sizes defined as small (*g* ≤ 0.2), medium (0.2 < *g* ≤ 0.8) and large (*g* > 0.8).

**Table 3.** Qualitative interpretation of the effect of the high principal components (PCs 1−3) on the symptomatic and control groups in the main feature sets for the pivot and non-pivot limb scenarios.

feature	mode(s) of variation^a^	phase(s) with greatest effect^b^	symptomatic group: nearest quartile^c^	interpretation of effect of feature on the symptomatic group: the symptomatic group tends to have…^d^
pivot limb				
*θ_HIPFLEX_* PC1	translation	step-down pivot	lower	more extended hip but same range of motion through step-down and pivot
*θ_HIPFLEX_* PC3	temporal shift	step-forward	lower	delayed initiation of hip flexion
*θ_KNEEEXT_* PC1	scaling	step-down pivot	lower	reduced peak and range of knee flexion through step-down and pivot
*M_ANKLEDF_* PC1	scaling	pivot	lower	increased peak and range of ankle plantarflexion moments
non-pivot limb				
*θ_ANKLEDF_* PC2	scaling temporal shift	step-down	lower	reduced peak and range of ankle dorsiflexion with delayed action
*M_HIPFLEX_* PC2	scaling	step-down step-forward	lower	reduced peak and range of hip flexion moments through step-down, and initiation of step-forward
*M_HIPADD_* PC3	scaling temporal shift	pivot	upper	delayed hip adductor action in pivot; lower peak and range of moments through pivot
*M_HIPROT_* PC1	scaling	step-down	upper	reduced peak and range of hip rotator moments
*M_KNEEEXT_* PC2	temporal shift	step-down	lower	delayed initiation of knee flexor moments prior to pivot phase

^a^ Effect(s) of the principal component (PC) on the original waveform (*scaling, translation* or *temporal shift*).

^b^ The temporal window(s) during which the PC had greatest effect on the waveform of the original variable (*step-down, pivot* or *step-forward*).

^c^ Indicates whether the mean principal component score for the symptomatic group is nearer the upper or lower quartile of principal component scores for the pooled data, based on the sign of the principal component scores for each group (table 2).

^d^ Qualitative interpretation of the effect of the PC on the symptomatic group with respect to the biomechanics of the step-down-and-pivot task.

## 3. Results

Female participants accounted for 30% and 18% of the symptomatic and control groups, respectively, and 72% of symptomatic participants presented with bilateral hip/groin pain. The symptomatic group reported significantly worse outcomes for all iHOT-33 and HAGOS measures compared with controls (table 1).

### 3.1. Between-group comparison of original biomechanical variables

**Figure 2.**
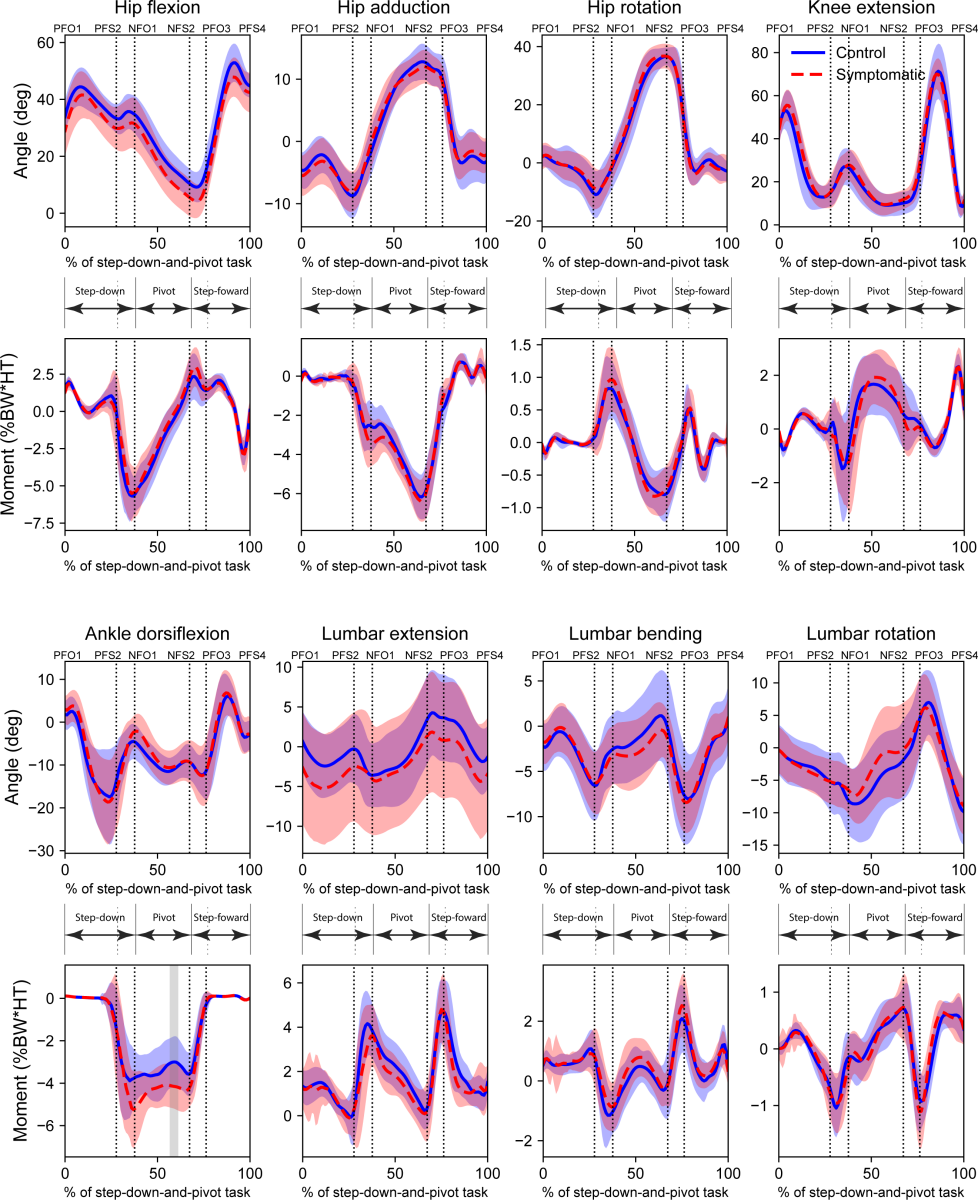
Pivot limb joint angles and moments for the step-down-and-pivot task for symptomatic and control groups. Ensemble waveforms were compared using *t*-tests with statistical parametric mapping. Grey shaded regions represent regions of statistical significance (α=0.003125). Task phases: Step-down; Pivot; and Step-forward. Event labels have format *[Limb][Event type][Sequence number]. Limb*: P (pivot) or N (swing); *Event type*: FO (foot-off) or FS (foot-strike). E.g. PFO3 is a foot-off on the pivot limb which is the third event on the pivot limb.

**Figure 3.**
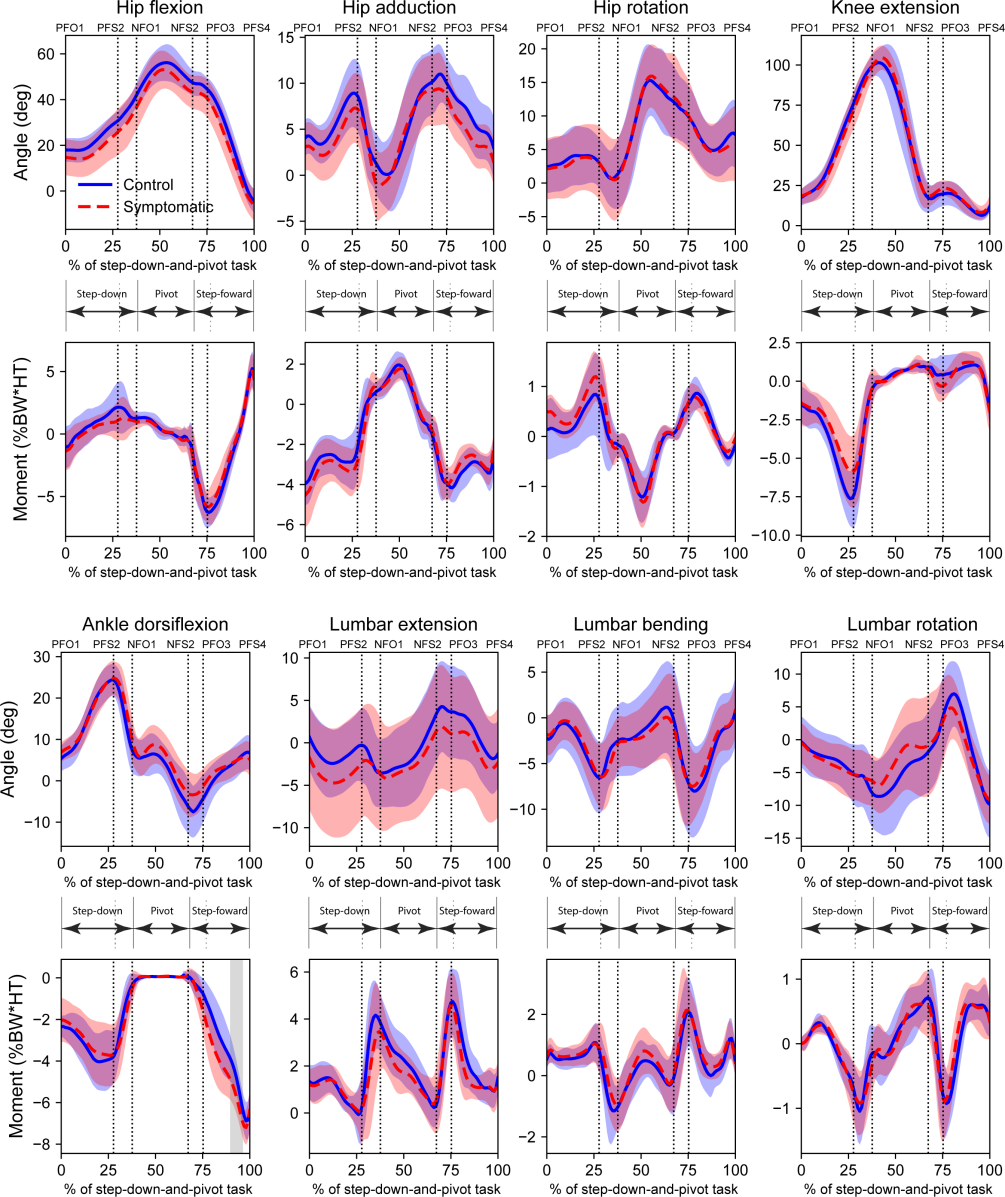
Swing limb joint angles and moments for the step-down-and-pivot task for symptomatic and control groups. Ensemble waveforms were compared using *t*-tests with statistical parametric mapping. Grey shaded regions represent regions of statistical significance (α=0.003125). Task phases: Step-down; Pivot; and Step-forward. Event labels have format *[Limb][Event type][Sequence number]. Limb*: P (pivot) or N (swing); *Event type*: FO (foot-off) or FS (foot-strike). E.g. PFO3 is a foot-off on the pivot limb which is the third event on the pivot limb.

No statistically significant differences were found between the symptomatic and control groups for any of the eight joint angles and moments of the pivot limb (figure 2), except for a brief period of significance in ankle dorsiflexion moment towards the end of the pivot phase. Similarly, no statistically significant differences were found between symptomatic and control groups for any of the eight joint angles and moments of the swing limb, except for brief periods of significance in ankle dorsiflexion moment during the step-forward phase (figure 3). Full SPM curves for these results are presented in electronic supplementary material, figures S2 and S3.

### 3.2. Analysis of biomechanical features of the pivot limb

**Figure 4.**
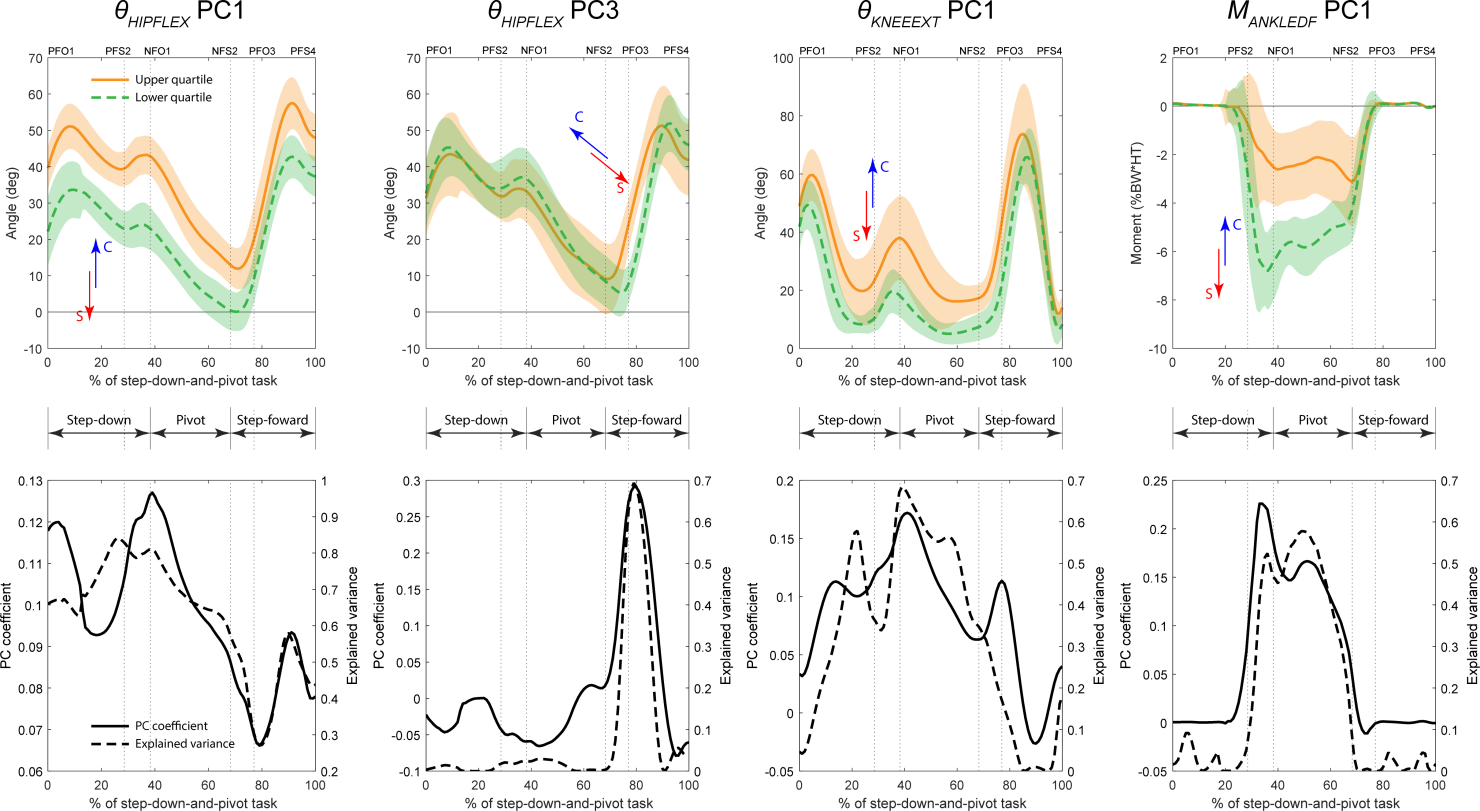
High principal components (i.e. PC1−3) from the final set of main features on the pivot limb. Top row: waveforms of the pooled original data representing the upper (solid orange) and lower (dashed green) quartiles of the principal component scores for each respective feature. Arrows signify the tendency for symptomatic (red) or control (blue) groups to be associated with the upper or lower quartile, e.g. for *θ_HIPFLEX_* PC1, the control group tended towards the upper quartile, while the symptomatic group tended towards the lower quartile. Bottom row: waveforms of the principal component coefficients (solid black) and variance in the original data explained by that principal component coefficient (dashed black). Task phases: Step-down; Pivot; and Step-forward. Event labels have format *[Limb][Event type][Sequence number]. Limb*: P (pivot) or N (swing); *Event type*: FO (foot-off) or FS (foot-strike). E.g. PFO3 is a foot-off on the pivot limb which is the third event on the pivot limb.

Despite the lack of statistically significant group differences in the original biomechanical variables, all final features were able to discriminate between symptomatic and control biomechanics at significance level α=0.05 (table 2). Six of the 10 final features of the pivot limb were able to discriminate between groups at the more conservative significance level α=0.001. Of these, four were high PCs: *θ_HIPFLEX_* PC1 (read as *‘the first principal component of hip flexion angle’*), *θ_HIPFLEX_* PC3, *θ_KNEEEXT_* PC1 and *M_ANKLEDF_* PC1 (figure 4, table 3), explaining 63.2%, 8.4%, 28.5% and 33.5% of the variance in their respective original variables (electronic supplementary material, table S1). For all four high PCs, the symptomatic group was more closely associated with the lower quartile of the PC scores for the pooled original data. These high PCs had greatest effect, and therefore showed the largest group differences, during the step-down (*θ_HIPFLEX_* PC1, *θ_KNEEEXT_* PC1) and pivot phases (*θ_HIPFLEX_* PC1, *θ_KNEEEXT_* PC1, *M_ANKLEDF_* PC1), with only *θ_HIPFLEX_* PC3 acting during the step-forward phase (figure 4, table 3). They described three modes of variation with respect to their respective original variables: translation (*θ_HIPFLEX_* PC1), scaling (*θ_KNEEEXT_* PC1, *M_ANKLEDF_* PC1) and temporal shift (*θ_HIPFLEX_* PC3). The qualitative interpretation of these features and the modes of variation they describe in the context of the step-down-and-pivot task are given in table 3.

### 3.3. Analysis of biomechanical features of the swing limb

**Figure 5.**
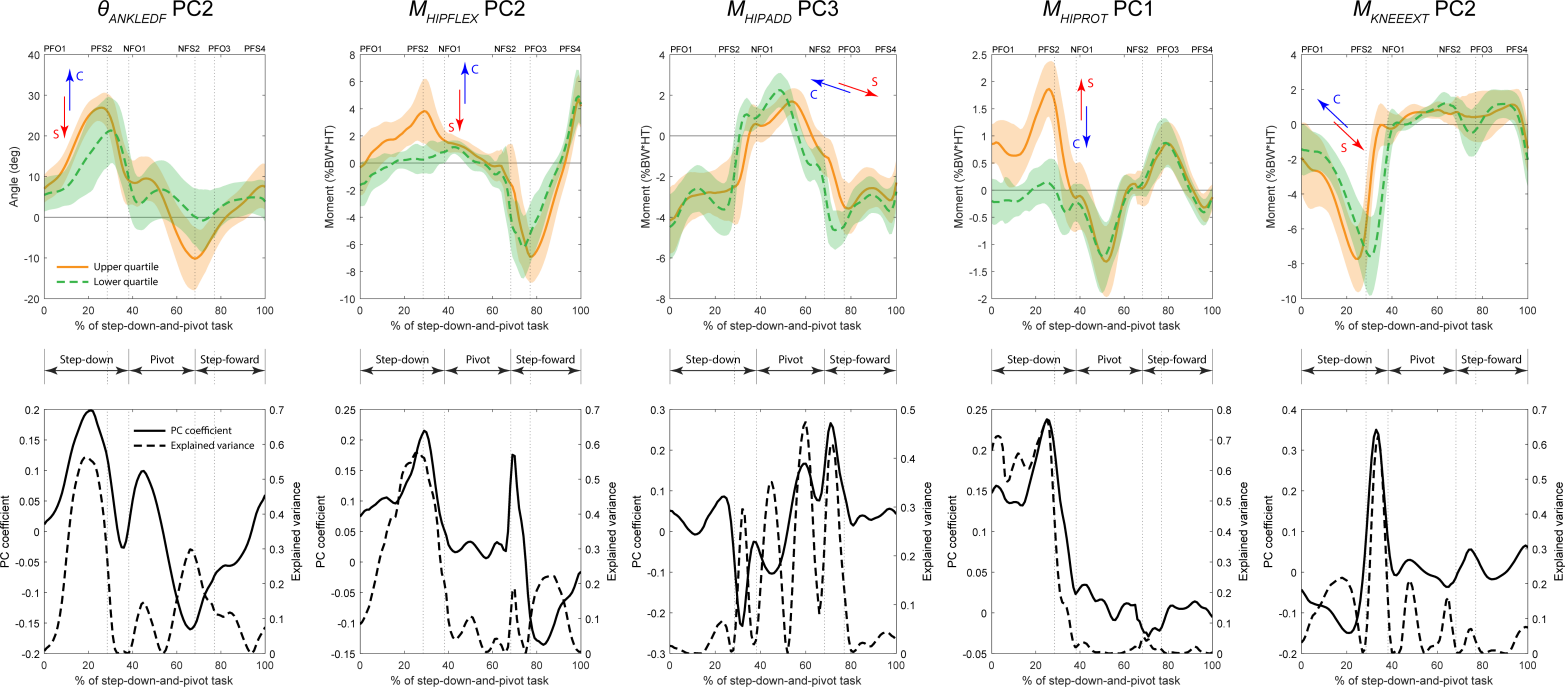
High principal components (i.e. PC1−3) from the final set of main features on the swing limb. Top row: waveforms of the pooled original data representing the upper (solid orange) and lower (dashed green) quartiles of the principal component scores for each respective feature. Arrows signify the tendency for symptomatic (red) or control (blue) groups to be associated with the upper or lower quartile, e.g. for *M_HIPROT_* PC1, the control group tended towards the lower quartile, while the symptomatic group tended towards the upper quartile. Bottom row: waveforms of the principal component coefficients (solid black) and variance in the original data explained by the respective principal component coefficient (dashed black). Task phases: Step-down; Pivot; and Step-forward. Event labels have format *[Limb][Event type][Sequence number]. Limb*: P (pivot) or N (swing); *Event type*: FO (foot-off) or FS (foot-strike). E.g. PFO3 is a foot-off on the pivot limb which is the third event on the pivot limb.

Only one of the 10 final features of the swing limb (*M_HIPROT_* PC1) was able to discriminate between groups at the more conservative significance level α=0.001 (table 2). Five features were high PCs: *θ_ANKLEDF_* PC2, *M_HIPFLEX_* PC2, *M_HIPADD_* PC3, *M_HIPROT_* PC1, *M_KNEEEXT_* PC2 (figure 5, table 3), explaining 18%, 19.9%, 12.1%, 29% and 19% of the variance in their respective original variables (electronic supplementary material, table S2). The symptomatic group was associated with the lower quartile of the PC scores for the pooled original data for three high PCs (*θ_ANKLEDF_* PC2, *M_HIPFLEX_* PC2, *M_KNEEEXT_* PC2), and the upper quartile for two high PCs (*M_HIPADD_* PC3, *M_HIPROT_* PC1). These PCs had the greatest effect, and therefore showed the largest group differences, during the step-down (*θ_ANKLEDF_* PC2, *M_HIPFLEX_* PC2, *M_HIPROT_* PC1, *M_KNEEEXT_* PC2), pivot (*M_HIPADD_* PC3) and step-forward (*M_HIPFLEX_* PC2) phases (figure 5, table 3).

### 3.4. Analysis of associated features

When distinguishing between symptomatic and control groups at significance level α=0.05, two significant associated features were found on the pivot limb, while five were found on the swing limb (table 4). Of these, two were able to distinguish between groups at the more conservative significance level α=0.001. The full set of associated features is provided as electronic supplementary material, tables S3 and S4.

**Table 4.** Main features that also have significant associated features.

main feature	significant associated features	*ρ*	principal component scores			
			symptomatic	control	*p*	*g*
pivot limb						
*θ_HIPFLEX_* PC3	*θ_KNEEEXT_* PC2	0.573	4.02 (46.6)	−13.5 (48.2)	0.002	0.370
*θ_HIPFLEX_* PC6	*θ_KNEEEXT_* PC5	−0.646	−1.47 (25.3)	6.35 (23.3)	0.009	−0.317
swing limb						
*M_HIPFLEX_* PC2	*M_KNEEEXT_* PC1	−0.575	0.856 (9.67)	−3.62 (7.75)	<0.001	0.492
*M_HIPROT_* PC1	*M_HIPADD_* PC1	−0.621	−0.597 (6.35)	2.31 (6.19)	<0.001	−0.461
*M_KNEEEXT_* PC2	*θ_KNEEEXT_* PC1	−0.572	4.27 (68.9)	−16.0 (106)	0.042	0.242
	*M_ANKLEDF_* PC3	0.557	−0.179 (3.81)	0.898 (4.33)	0.024	−0.269
	*M_LUMBAREXT_* PC3	0.681	−0.222 (4.47)	0.960 (5.87)	0.048	−0.236

Only significant associated features are shown, defined as associated features that satisfied two conditions: (i) moderate, 0.5≤|ρ|<0.7, or strong. |ρ|≥0.7, correlation with their respective main feature at significance α=0.05; and (ii) group differences in principal component scores significant at level t≥2.0 (approximately, α=0.05).

Effect sizes for principal component scores were calculated using Hedge’s *g*.

## 4. Discussion

Our objective was to quantify and compare differences in biomechanics between athletes with and without hip/groin pain when undertaking a multi-directional functionally relevant movement task. Specifically, we aimed to identify and describe the biomechanical features that differentiate the two groups during a step-down-and-pivot task. We found some small, albeit significant, differences in ankle joint mechanics during the task, but overall few differences were evident irrespective of whether the ‘reference’ limb was the pivot or swing limb. Nevertheless, by means of our feature selection process based on PCA and statistical learning, we found two sets of 10 main features that were able to distinguish between symptomatic and control biomechanics during the step-down-and-pivot task with *p* < 0.05.

We developed our exploratory step-down-and-pivot task with the intention that both pivot and swing limbs would experience large hip joint forces during single support, as well as the combined movements of hip adduction and internal rotation when weight-bearing (e.g. the pivot limb during the pivot phase) and hip flexion, adduction and internal rotation when non-weight-bearing (e.g. the swing limb during the pivot phase). However, our findings of no meaningful differences for most biomechanical variables during the step-down-and-pivot task, despite the presence of considerable hip/groin pain in the symptomatic group, is consistent with our previous findings for this cohort undertaking tasks designed to provoke the hip [9,10]. Biomechanical studies of other hip/groin pain cohorts also found modest differences compared with asymptomatic controls [11,13,14,16,17,20,21,45–47]. The lack of meaningful biomechanical differences in our present cohort was previously attributed to the highly trained nature of our athletic participants, who were able to tolerate discomfort without substantially adapting their movement patterns [10], as well as the possibility that the investigated tasks did not challenge the hip sufficiently to elucidate a measurable response [9]. Unfortunately, our findings suggest these arguments may also reasonably apply to our present step-down-and-pivot task. As we did not ask participants to report the level of pain during the task, we were unable to truly assess how well our task could provoke pain in the symptomatic hip. Furthermore, the very small magnitude of differences in the original biomechanical variables between symptomatic and control groups on both the pivot and swing limbs could reflect the constrained nature of the task. That is, it is likely that the task objective strongly defined the overall movement pattern, with a limited allowance for large adaptations in joint angles or moments in response to pain, if any. Despite emulating more real-world activities, this may be one drawback of using multi-directional tasks for biomechanical analyses.

Nevertheless, emerging evidence from biomechanics studies after knee injury suggests that small aberrations in movement patterns may be associated with conditions such as post-traumatic osteoarthritis [48–51]. This is particularly relevant to our symptomatic cohort, which comprises mostly physically active young adults [23] (table 1) who may be at risk of developing future hip osteoarthritis before middle age [52,53]. Therefore, the capacity to identify and describe subtle biomechanical differences early in the disease process may be crucial for implementing effective monitoring and developing potential future interventions. With our feature selection process, we were able to isolate and amplify these subtle biomechanical adaptations during the step-down-and-pivot task, and discriminate between groups with *p* < 0.05. Importantly, we found differences similar to those of a PCA-based study of level walking individuals with more advanced hip osteoarthritis [33], including evidence of decreased hip and knee extension angles and altered hip flexion and internal rotation moments, demonstrating the discriminatory power of our approach.

The presence of hip/groin pain had a subtle, but notable impact on the biomechanics of the step-down-and-pivot task that agrees with reported adaptations for similar cohorts in more routine linear tasks [18] such as walking, stair ascent and sit-to-stand. With respect to the pivot limb, symptomatic individuals undertook the step-down (non-weight-bearing) and pivot (weight-bearing) phases with a straighter lower limb, but with subsequent delayed initiation of the step-forward phase. Specifically, they stepped down with a more extended hip compared with control individuals (pivot limb *θ_HIPFLEX_* PC1, figure 4), a characteristic that continued through the pivot phase. In fact, PCs associated with hip angles accounted for 5 out of 10 main features on this limb. This was accompanied by a reduced flexion angle and total range of motion at the knee throughout the step-down and pivot phases (pivot limb *θ_KNEEEXT_* PC1, figure 4). Despite the constrained nature of the task, these small adaptations may help mitigate some pain during the high loading experienced during foot strike at the end of the step-down phase, as well as any discomfort associated with hip adduction and internal rotation during the pivot phase. However, we found that one consequence of this approach was higher demand from the ankle plantar flexor muscles during the pivot phase (pivot limb *M_ANKLEDF_* PC1, figure 4), a statistically significant finding evident in the original ankle moments (figure 2). This may be due to the greater challenge of balancing and coordinating the pivoting action with a straighter lower limb, which has impacts for the ground force vector and foot-ground centre-of-pressure, a characteristic frequently reported for hop landings after injury [54]. Thus, small differences in the original biomechanical variables at the hip and knee were accompanied by large, probably compensatory, differences at the ankle on the pivot limb in the symptomatic group (figure 2). Furthermore, we found that symptomatic individuals delayed the initiation of the step-forward phase (pivot limb *θ_HIPFLEX_* PC3, figure 4), suggesting a reluctance to move the hip into flexion. To our knowledge, delayed action is a mode of variation not previously reported in biomechanical studies of hip/groin pain, and, albeit small, should be considered when evaluating symptomatic individuals in the clinical setting.

With respect to the swing limb, no high PCs associated with hip angles could distinguish between symptomatic individuals and controls; however, hip moments figured prominently, accounting for 5 out of the 10 main features on this limb. Symptomatic individuals tended to utilize a smaller hip flexor moment (swing limb *M_HIPFLEX_* PC2, figure 5) during the step-down (weight-bearing) phase as well as the initiation of step-forward (weight-bearing), while demonstrating delayed hip adductor action through the pivot phase (swing limb *M_HIPADD_* PC3, figure 5). These reflect broad agreement with our findings for the pivot limb. However, the symptomatic limb also developed a greater hip internal rotator moment during the step-down phase (swing limb *M_HIPROT_* PC1, figure 5), which occurred together with greater hip abductor moment (negative PC score for swing limb *M_HIPADD_* PC1, a significant associated feature of *M_HIPROT_* PC1, table 4). These differences were large enough to be evident in the original variables, albeit not statistically significant (figure 3). The primary hip abductor, gluteus medius, also externally rotates the hip, implying elevated hip rotator coactivation. Thus, it follows that symptomatic individuals probably found a greater need to control the weight-bearing swing limb while stepping down, to possibly avoid the kinematic configurations that exacerbate pain in this cohort [9,55], as evidenced by reduced hip angles (figure 3).

From our findings discussed thus far, we observed that the highest PCs in each main feature set (i.e. PCs 1−3 of the selected variables; four on the pivot limb; five on the swing limb) described just three basic modes of variation: (i) scaling; (ii) translation; and (iii) temporal shift, that were easily interpretable into the present biomechanical context. These can be illustrated using the simple analogy of a generalized linear function, whereby a segment of the mean waveform *y* of a variable within a time window $t\in \left[t_{0},t_{1}\right]$ is described by some function *f(t)*,


(4.1) $$y=f\left(t\right),\;\;t_{0}\leq t\leq t_{1}.$$

Each of the three modes of variation can be envisaged by adding terms to equation (4.1) as follows:


(4.2) $$y=Af\left(t-C\right)+B,\;\;t_{0}\leq t\leq t_{1},$$

where *A* represents a scaling factor, *B* is a translation offset and *C* is a temporal offset, with these terms able to take positive or negative values. We can see from equation (4.2) that:

(1) *Scaling* inflates or deflates both the peaks and range of the waveform, stretching or flattening the waveform, respectively (e.g. pivot limb *M_ANKLEDF_* PC1, figure 4).
(2) *Translation* shifts the waveform up or down, increasing or decreasing peaks, but leaving the waveform shape and range unchanged (e.g. pivot limb *θ_HIPFLEX_* PC1, figure 4).
(3) *Temporal shift* offsets the waveform forward or back in time, resulting in delayed or pre-emptive action, respectively (e.g. swing limb *θ_KNEEEXT_* PC1, figure 5).

As we normalized all waveforms to 101 samples, our present study did not identify a fourth potential mode of variation—temporal scaling—in which the waveform segment is stretched or compressed. PCA is a within-sample analysis, yet because the 10 high PCs in our main feature sets described up to 63% of the variance of their respective variables (electronic supplementary material, tables S1 and S2), they are likely to be features of step-down-and-pivot found in the population overall. Although PCs are abstract concepts, as shown thus far, the modes of variation the high PCs expressed were simple to describe and, therefore, highly interpretable in the context of our present study. On the contrary, the remaining 10 main features were lower PCs describing at most 9% of the variation of their respective biomechanical variables (electronic supplementary material, tables S1 and S2). They are more likely to be sample-specific, thus reflecting the overall small between-group differences in biomechanical variables. Therefore, these low PCs are less useful for understanding the impact of hip/groin pain on step-down-and-pivot biomechanics more broadly.

Our present study demonstrates the merit of investigating multi-phase tasks in biomechanical studies of hip/groin pain. We found that, for many of the high PCs in our main feature sets, the explained variance peaked at or near the key transitions within the task (step-down to pivot, and pivot to step-forward), indicating that these PCs had the greatest effect on the waveforms of their respective variables during these time points (figures 4 and 5). Therefore, the largest group differences discussed thus far also occurred during these transitions. While more routine linear tasks, such as walking, running, squatting and stair climbing among others [18], have been well-studied in the context of hip/groin pain, to our knowledge, few studies have investigated tasks with multiple components, such as the single-leg drop-jump [10] and cutting manoeuvres [15], and therefore no known previous study has specifically reported biomechanical differences at the transitions between task components. However, as previously discussed, such tasks may be more constrained in their nature. Thus, it is important to reiterate that the true differences in joint angles and moments between groups in our present study were small, with qualitative interpretation only possible because our feature selection process was able to isolate and amplify these differences. Nevertheless, movement routines developed as physical interventions for hip/groin pain may benefit from including complex movements and emphasizing transitions between movement phases.

Our study was not without limitations. Firstly, most of the individuals in our symptomatic group reported bilateral rather than unilateral pain and/or symptoms (table 1), which was a feature of the FORCe cohort more broadly [9,10]. For our present study, we only considered the more symptomatic limb for analysis. In fact, the less symptomatic limb also showed small biomechanical differences compared with controls that were similar to the more symptomatic limb (electronic supplementary material, figures S2–S7). With respect to mechanical dynamics, it is unlikely that the less symptomatic limb affected the motion of the more symptomatic limb in a meaningful way, as mechanical contributions from the contralateral limb to ipsilateral limb mechanics are typically very small in low-speed non-ballistic tasks such as walking [56]. However, we cannot discount entirely that pain and/or symptoms in the less symptomatic limb may have influenced neuromotor adaptations in the more symptomatic limb [57]. Thus, our findings should be interpreted in the light of this limitation.

Secondly, experimental biomechanical data is trimodal, i.e. *variable versus trial versus time*; however, PCA can only effectively examine bimodal data. In our present analysis, we independently analysed trial versus time for each biomechanical variable. We subsequently used our feature selection process, which is based on robust, reliable and transparent statistical learning methods, to contend with relationships between variables, particularly the use of sequential feature selection to ensure the selection of uncorrelated variables in the final feature sets. A multi-modal analysis method, such as parallel factor analysis (PARAFAC), would preclude the need for our lengthy multi-step feature selection process. However, techniques such as PARAFAC can be more computationally expensive than PCA, and may not be able to guarantee unique solutions due to the non-convex nature of their solution strategies.

Thirdly, our present study could not examine sex differences in step-down-and-pivot biomechanics within groups and between groups. Our previous studies using the broader FORCe cohort have shown that sex may be an important effect modifier for individuals with hip/groin pain in this cohort, in both high- and low-impact activities [19,58]. However, only a small subset of the FORCe cohort undertook the step-down-and-pivot task, with the number of female participants insufficient to examine sex differences with enough statistical power (table 1).

Finally, although our objective was to compare only two groups—symptomatic and control limbs—our analysis involved many independent statistical tests applied to the numerous PCs at every stage of our pipeline. As we did not specifically adjust our significance levels to account for multiple tests, it suggests the possibility of increased type 1 error, and therefore the selection of spurious features in our final sets. However, our systematic multi-step approach was designed to minimize this possibility, in particular, our implementation of a naive Bayes classification scheme with 10-fold cross-validation repeated 1000 times. Gait studies using PCA with naive Bayes classifiers have been shown to achieve 0.99 specificity [35]. Therefore, we are confident that the chance of any spurious features appearing in our final sets is minimal. Furthermore, in testing group differences in each of the final feature sets (table 2), we could have applied a Bonferroni-corrected α=0.005 to account for the 10 multiple comparisons in each set. However, we adopted a more cautious approach, performing inference at a significance level of α=0.001 as the final features were already significant between groups at α=0.05. Thus, our final features represent highly conservative results, in which we have, to the best of our ability, reduced the possibility of type 1 error. All things considered, our present study is exploratory, and having identified a candidate set of features that could distinguish between symptomatic and control biomechanics for the step-down-and-pivot task, a future study could re-analyse these features with even greater statistical rigour.

## 5. Conclusion

In conclusion, differences in the lower-limb joint angles and moments of a novel step-down-and-pivot task between individuals with hip/groin pain and asymptomatic controls were small. However, using a feature selection process based on PCA and statistical learning methods, we were able to quantify and describe these differences, identifying three distinct modes of variation at the hip, knee, ankle and lumbosacral joints. Our findings highlight the merit of examining three-dimensional multi-phase tasks as key differences occurred at transitions between task components. Most importantly, our study may help understand the role of small but persistent aberrations in lower-limb biomechanics in the development of future osteoarthritis, and the inclusion of multi-phase tasks may benefit the design of movement routines for intervention.

## Data Availability

The Matlab code for implementing our feature selection pipeline is available via Zenodo [59]. All data essential to this study is also available via Zenodo [60]. The supplied data is fully de-identified. Participant and demographic information associated with the FORCe project are sensitive and not automatically provided. To discuss the use of participant and demographic data, please contact the FORCe programme director Prof. Kay Crossley, k.crossley@latrobe.edu.au, or the La Trobe Sports & Exercise Medicine Research Centre, lasem@latrobe.edu.au. Supplementary material is available online [61].
